# Dietary Choline Intake: Current State of Knowledge Across the Life Cycle

**DOI:** 10.3390/nu10101513

**Published:** 2018-10-16

**Authors:** Alejandra M. Wiedeman, Susan I. Barr, Timothy J. Green, Zhaoming Xu, Sheila M. Innis, David D. Kitts

**Affiliations:** 1 BC Children’s Hospital Research Institute, Vancouver, BC V5Z 4H4, Canada; awiedeman@bcchr.ca (A.M.W.); tim.green@sahmri.com (T.J.G.); 2 Food, Nutrition, and Health Program, The University of British Columbia, Vancouver, BC V6T 1Z4, Canada; susan.barr@ubc.ca (S.I.B.); zhaoming.xu@ubc.ca (Z.X.); 3 South Australia Health and Medical Research Institute, Adelaide, SA 5000, Australia

**Keywords:** choline, dietary choline forms, human milk, breast milk, dietary recommendations, adequate intake, dietary assessment

## Abstract

Choline, an essential dietary nutrient for humans, is required for the synthesis of the neurotransmitter, acetylcholine, the methyl group donor, betaine, and phospholipids; and therefore, choline is involved in a broad range of critical physiological functions across all stages of the life cycle. The current dietary recommendations for choline have been established as Adequate Intakes (AIs) for total choline; however, dietary choline is present in multiple different forms that are both water-soluble (e.g., free choline, phosphocholine, and glycerophosphocholine) and lipid-soluble (e.g., phosphatidylcholine and sphingomyelin). Interestingly, the different dietary choline forms consumed during infancy differ from those in adulthood. This can be explained by the primary food source, where the majority of choline present in human milk is in the water-soluble form, versus lipid-soluble forms for foods consumed later on. This review summarizes the current knowledge on dietary recommendations and assessment methods, and dietary choline intake from food sources across the life cycle.

## 1. Introduction

**Figure 1:**
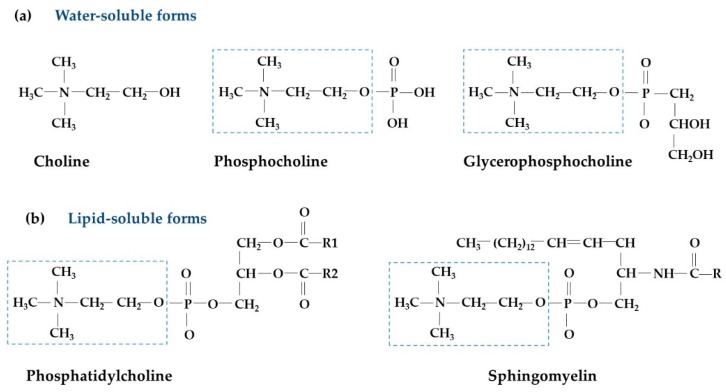
Structures of different choline forms: (**a**) Water-soluble forms; (**b**) lipid-soluble forms. Dashed box indicates free choline, R represents a fatty acid chain.

Choline (2-hydroxyethyl-trimethyl-ammonium salt; molecular weight of 104 g/mol) is an essential nutrient for humans. While choline can be obtained through endogenous synthesis [1], this is not normally enough to support body needs. As such, choline needs to be obtained from the diet [2]. Choline has important and diverse functions in both cellular maintenance and growth across all life stages, including roles in neurotransmission, membrane synthesis, lipid transport, and one-carbon metabolism [3,4,5]. Signs of choline deficiency have been reported in humans fed experimental choline-deficient diets and also in patients receiving total parenteral nutrition [6,7]. In 1998, the Institute of Medicine recognized choline as an essential nutrient and established dietary intake recommendations [8]. Similarly, the European Food Safety Authority set dietary recommendations for choline in 2016 [9]. In foods, choline is found as both water-soluble (free choline, phosphocholine, and glycerophosphocholine) and lipid-soluble forms (phosphatidylcholine and sphingomyelin) (Figure 1) [2]. 

Different forms of choline vary in how absorption and metabolism occur. After absorption, water-soluble forms of choline reach the liver through portal circulation while lipid soluble forms are packaged into chylomicrons, which are absorbed and transported through lymphatic circulation [10]. Interestingly, the different dietary choline forms consumed during infancy differ from those in adulthood. This can be explained by the primary food source, where the majority of choline present in human milk is in the water-soluble form, versus lipid-soluble forms for foods consumed later on. Hence, it has been suggested that the form in which dietary choline is consumed should be considered [11]. Evidence from animal studies have shown that different forms of choline present in milk will be utilized differently, as evidenced by the fact that maternal and offspring immune systems respond differently to various forms of choline consumed [12,13,14,15]. Therefore, the intake of different forms of choline should be considered relevant at specific stages of development. Here, we review the current knowledge on choline food composition, dietary recommendations, dietary assessment methods, and dietary choline intake across the life cycle. A brief section on metabolism and biological functions of choline is also included to provide context to the different forms of choline discussed in the review. 

## 2. Overview of Choline Metabolism and Biological Functions

### 2.1. Choline Metabolism

**Figure 2:**
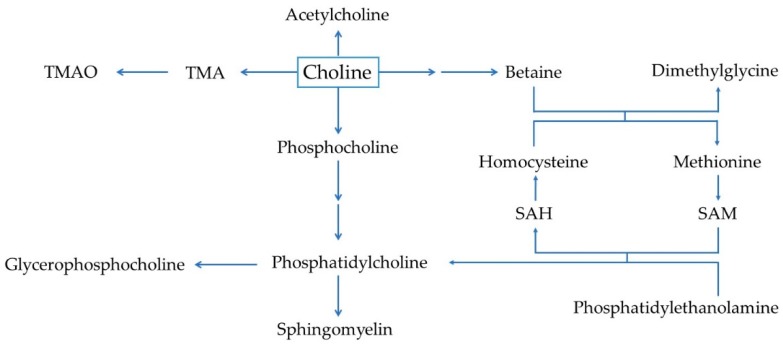
Simplified overview of choline metabolism. Abbreviations: SAM, *S*-adenosylmethionine; SAH, *S*-adenosylhomocysteine; TMA, trimethylamine; TMAO, trimethylamine-*N*-oxide.

Choline metabolism can be divided into four main pathways which are involved in the synthesis of acetylcholine, trimethylamine (TMA), betaine, and phospholipids (Figure 2). Choline is used as the precursor for the synthesis of the neurotransmitter, acetylcholine, by choline acyltransferase in the cytosol of pre-synaptic cholinergic neurons [16]. Acetylcholine is subsequently packaged into vesicles and released into the synaptic cleft, where it binds to receptors of the post-synaptic neuron in the central and peripheral nervous systems [17]. Acetylcholine synthesis has also been reported in tissues, including placenta, muscle, intestine, and lymphocytes [18,19]. In the large intestine, choline is metabolized to TMA by the gut microbiota prior to absorption [20,21]. After absorption, TMA is metabolized to trimethylamine-*N*-oxide (TMAO) by flavin monooxygenases in the liver [22].

Choline can be irreversibly oxidized to yield betaine in a two-step process catalyzed by choline dehydrogenase and betaine aldehyde dehydrogenase mainly in the liver and kidney [23,24]. Betaine is an important osmolyte and a methyl group donor. As a methyl group donor, betaine participates in the re-methylation of homocysteine to methionine by betaine-homocysteine *S*-methyltransferase (BHMT), also producing dimethylglycine [25,26]. This reaction is an alternative pathway, parallel to the ubiquitous vitamin B12-folate-dependent pathway for homocysteine re-methylation [27]. The BHMT accounts for up to half of the hepatic homocysteine re-methylation activity [28]. Methionine is the precursor of the universal methyl donor, *S*-adenosyl methionine (SAM), which is involved in several methylation reactions, such as epigenetic regulation of DNA as well as the synthesis of phosphatidylcholine [29,30]. As for betaine, dimethylglycine synthesis occurs primarily in the liver and kidney [31,32,33], and further demethylation of dimethylglycine produces sarcosine, which is subsequently metabolized to glycine, resulting in a carbon unit transferred to the folate pool [34]. 

Finally, choline is a precursor for the synthesis of phosphatidylcholine, the most abundant form of phospholipid in the body. Phosphatidylcholine is synthesized through the cytidine diphosphate (CDP)-choline pathway, which occurs in all nucleated cells [35]. It has been estimated that 70% of total phosphatidylcholine in the liver is synthesized by this pathway [36,37,38]. Alternatively, phosphatidylcholine can be generated by the de novo synthesis pathway by the sequential methylation of phosphatidylethanolamine by phosphatidylethanolamine *N*-methyltransferase (PEMT) [39,40,41]. This reaction consumes three molecules of SAM, which in turn generate three molecules of *S*-adenosyl homocysteine (SAH), a precursor of homocysteine [1,42]. It has been estimated that up to 50% of homocysteine production may originate from PEMT activity, with the highest activity being detected in the liver (although activity is also observed in other tissues, such as the mammary gland) [1,37,39,43,44,45,46]. In humans, this is the only known endogenous de novo pathway for choline synthesis. Recently, it has been reported that phosphatidylcholine produced by the PEMT pathway differs from that originating from the CDP-choline pathway, particularly in the fatty acid composition, with the first characterized by having a higher composition of long-chain fatty acids, such as docosahexaenoic acid [47,48]. 

### 2.2. Biological Functions of Choline 

Choline has received considerable attention due to its inverse association with adverse health outcomes that can occur across the life cycle, including birth defects, neurodevelopment and cognition alterations, hepatic steatosis, cardiovascular disease (CVD), and cancer [5,7,49,50,51,52,53,54,55,56,57,58,59]. Oxidation of choline to betaine and subsequent SAM synthesis are critical methylation reactions that represent a cornerstone for epigenetic regulation of gene expression [60,61]. In rodents, maternal choline-deficient diets during the perinatal period altered DNA and histone methylation in the offspring [62,63,64]. In humans, low maternal choline intake during pregnancy can alter DNA methylation in the placenta and cord blood [65]. Notably, there is an inverse relationship between the risk of neural tube defects and maternal choline intake or plasma choline concentrations, independent of dietary folate or supplemental folic acid intakes [49,52]; to some extent, this is analogous to that reported for folate. In addition, other birth defects associated with choline deficiency include cleft lip, hypospadias, and cardiac defects [66,67,68,69].

The role of choline in neurodevelopment and cognition involves not only the synthesis of acetylcholine and components of cellular membranes, but also gene expression. In rodents, maternal choline intake during the perinatal period impacts both anatomical and biochemical aspects of cognitive function, along with lifelong effects, including memory decline in the offspring as they age [70]. The neuroprotection effect of choline observed in animal studies has also been studied in humans; however, results are inconclusive [53,54,71,72,73]. In children, only two studies have been published, and no association was found between plasma free choline concentrations and child cognition, albeit plasma betaine concentrations were positively associated with language [55,74]. In adults, positive associations between cognition and plasma free choline concentrations, and between dietary choline intake and better cognitive performance, have been described [75,76]. However, other researchers examining choline supplementation, in adults, have reported inconsistent results [77,78,79,80,81,82]. Therefore, more research is required to clarify the relationship between choline and cognitive function in different age groups.

In humans, liver damage (e.g., elevated serum alanine aminotransferase concentration) occurred in healthy men after only three weeks of dietary choline restriction (*n* = 7, 0.42 to 0.62 µkat/L), which was not observed in the control group (*n* = 8, 0.40 to 0.32 µkat/L) [7]. In the same study, a 30% decrease in plasma free choline concentration was observed in the choline-deficient group. Similarly, muscular damage (e.g., elevated serum creatine phosphokinase concentration) was reported after three weeks of dietary choline restriction [83]. These examples of tissue damage were attributed to altered structural integrity and increased cellular membrane permeability that arises due to a decreased phosphatidylcholine to phosphatidylethanolamine ratio [84,85,86,87]. In addition, the production of very low-density lipoproteins requires phosphatidylcholine synthesis in the liver [88,89]. Without an adequate supply of choline for phosphatidylcholine synthesis, triacylglycerides will accumulate, which leads to fatty liver condition [90,91]. Similar alterations have been reported in patients receiving long-term total parenteral nutrition devoid of choline [92,93]. These data supported the classification of choline as an essential nutrient by indicating that de novo synthesis of choline is not sufficient to meet the body’s requirements in some instances.

The reported association between choline status and CVD risk is linked to homocysteine and TMAO concentrations; however, this area is not fully understood, and that evidence exists for pathways that could, at least in theory, either increase or decrease CVD risk. Elevated homocysteine concentrations have been positively associated with a risk of CVD [94,95]. In prospective cohort studies, dietary choline intakes were negatively associated with homocysteine concentrations, and plasma betaine concentrations were also negatively associated with risk of CVD [96,97]. In contrast, a recent meta-analysis reported no evidence of a positive association between dietary choline or betaine and CVD incidence [98]. Intervention studies, with betaine or phosphatidylcholine supplementation, have reported a reduction in homocysteine concentrations [99,100,101]. However, lowering homocysteine concentrations with B-vitamins, such as folate and B12, does not reduce CVD risk [102,103]. Furthermore, a concern about choline intake and CVD is related to a possible increase in TMAO concentration, which has been positively associated with CVD risk [104,105,106]. It has also been reported that only a low proportion of choline intake derived from eggs is converted to TMAO [107], which is then excreted and does not accumulate in the blood [108]. In addition to choline intake and gut microbiota, TMAO levels are also controlled by renal excretion [109]. To date, the mechanisms by which TMAO increases CVD risk and the identification of the type of bacteria involved in TMA synthesis are now becoming understood [21,110]. However, it is important to recognize that TMAO content is high in seafood [111], and only a small variation of TMAO concentrations can be explained by dietary intake [112].

## 3. Choline Content in Dietary Food Sources

### 3.1. Choline Concentration in Human Milk 

Human milk is the only source of choline for exclusively breastfed infants during the first six months of life, and is considered the optimal source of nutrition for infants by the World Health Organization [113]. Neonates and infants require large amounts of choline to support a rapid growth rate and optimal development [114]. It has been previously reported that total choline content in human milk increases from colostrum to two weeks after birth, and then stays stable beyond six months [115,116,117,118]. Studies have reported a total choline content in mature human milk ranging from 125 to 166 mg/L (1198 to 1600 µmol/L) (Table 1) [118,119]. In mature human milk, phosphocholine is the predominant form of choline, followed by glycerophosphocholine; thus, the water-soluble forms of choline account for approximately 84% of the total choline [115,116,120,121]. In contrast, the lipid-soluble forms of choline (phosphatidylcholine and sphingomyelin) are mainly present as a minor component of the milk fat globule membrane, and thus make up a relatively small fraction of the total choline in human milk [122,123]. Milk choline is either transported from the maternal circulation or obtained through de novo synthesis via the PEMT pathway in the mammary gland [124,125]. It has been described that the concentrations of total choline in human milk increase almost two-fold during the first week after birth and remain relatively constant thereafter in mature milk [115,116,126].

Some authors have suggested that maternal dietary choline intake may influence the milk choline concentration [127,132,133]. Choline supplementation studies have reported a significant increase in the concentrations of free choline, phosphocholine, glycerophosphocholine, and total choline (ranging between 20% and 38%) in mature milk [119,120]. Studies of the choline concentration in human milk have been reported mostly from high-income countries, where the consumption of food of animal origin, the richest source of choline, is presumably higher compared to low-income countries [134,135,136]. A small study comparing choline concentrations, including free choline, phosphatidylcholine, and sphingomyelin, in milk samples from lactating women in the US and Ecuador reported that women from Ecuador had a lower concentration of free choline than that in the US, but lipid-soluble forms did not differ [133]. This observation was attributed to a possible difference in dietary choline intake; however, the actual intake of choline was not assessed in the study. Furthermore, free choline represents only a small fraction of the total choline in human milk; thus, this difference may not be of biological relevance. Recently, we published the first report on concentrations of the water-soluble forms of choline in mature milk samples from lactating women in Canada and Cambodia [137]. Our results indicated that the concentrations did not differ between Canadian and Cambodian women. 

Different methodologies used for human milk sampling and determination of choline concentration have been used, and this fact is important in reviewing differences in population-based choline intakes of breast-fed infants. For example, milk samples have been collected as full breast expression as well as mid-feed samples, and the time of day of milk collection varies across studies. [138]. There is also range of different methodologies used to analyze milk choline, which include radio-enzymatic assay, detection using proton nuclear magnetic resonance, isolation and quantification using gas chromatography, and high-performance liquid chromatography coupled with mass spectrometry [119,121,126,127]. One advantage of using radiolabeled or stable isotopes is the allowance for simultaneous detection and analysis of choline metabolites [139,140,141]. Notwithstanding this, these differences in methodologies used in human milk collection and analyses of choline are potentially important factors to be aware of in comparing choline and metabolite concentrations among studies.

### 3.2. Choline Concentration in Infant Formulas

When breastfeeding is not possible, infant formulas are often used, and the nutrient content in mature human milk may be used as a guideline to develop the nutrient composition of human milk substitutes and enteral formulas for infants [142]. Current guidelines for total choline content in infant formulas recommend a minimum of 7 mg/100 kcal and a maximum of between 30 to 50 mg/100 kcal for choline content [143,144]. This range is equivalent to a total choline intake between 37 to 265 mg/day, based on a volume consumption of 0.78 L/day and energy content of 68 kcal/100 mL [143]. Although choline is included in infant formula, the individual choline forms can vary across different formulations and commercial brands, and certainly between formulas and different human milk samples [145]. Most of the commercially available infant formulas add choline as choline chloride and also may include a small amount from soy lecithin. Total choline content can vary up to two-fold in infant formulas, even among those formulas manufactured by the same manufacturer (52 to 104 mg/L) [146]. Commercial and hospital infant formulas available in different countries have been analyzed to have a total choline content ranging from 82 to 209 mg/L [147]. Studies conducted in the US reported that bovine milk-based formulas have lower free choline and phosphatidylcholine, with higher phosphocholine, glycerophosphocholine, and total choline content, compared to soy-based formulas [121,127]. When compared with human milk, bovine milk-based formulas had lower phosphocholine and sphingomyelin, and higher glycerophosphocholine, with a similar total choline content [121,127]. Later studies from the UK and Turkey have reported similar findings [115,116]. 

The significance of the different dietary choline forms has not been clearly explained at present. As mentioned before, studies in rodents have suggested that the different forms of choline vary in bioavailability and impact infant development, reflecting different absorption efficiencies and rates of tissue uptake [12,13]. Also, dietary phosphatidylcholine compared with free choline altered the form of choline present in milk, while the total choline concentrations in milk did not differ between groups [13]. However, in humans, the relationship between the different chemical forms of choline in milk and infant development is not well understood. There is only one study from Turkey reporting that exclusively breastfed infants have higher concentrations of free choline compared with formula-fed infants [116]. More research in this area is required to substantiate the difference in choline concentrations between different infant feeding practices at different intake levels. 

### 3.3. Choline Content in Dietary Food Sources 

The first database on the total choline and its individual forms, in foods that are common in North American diets, was made available in 2004 by the US Department of Agriculture (USDA). The data set listed 434 food items [148], which was updated and expanded in 2008 [2]. These databases include values for free choline, phosphocholine, glycerophosphocholine, phosphatidylcholine, and sphingomyelin, as well as values for total choline and betaine. Although betaine is a choline metabolite, it is generated by two successive irreversible reactions [149]; therefore, it is not a choline-containing molecule, nor can it be used to resynthesize choline [150]. Therefore, betaine is not included in the total choline value. However, dietary betaine may have a choline-sparing effect, particularly decreasing the use of choline used to synthesize betaine [151]. It is relevant to note that the first released version of the database for choline content in foods contained erroneously high betaine values, which were rectified in the second edition. This is important to mention when comparing betaine intake from studies using different available nutrient databases. Total choline, individual forms of choline, and betaine content in selected food sources are presented in Table 2.

Total choline content has been well documented to be higher in foods of animal origin, compared to foods of vegetable origin on a per unit of weight basis [2]. Foods that contain the highest content of choline include liver, eggs, beef, fish, pork, and chicken [2]. In these foods, the majority of choline is present as phosphatidylcholine, a lipid-soluble form, as part of the cell membrane. Milk is also a good food source for total choline, as it is usually consumed on a daily basis. Information on choline from specific food groups continues to be expanded, with recent data for choline content in pulses and meats becoming available from Canada [152,153]. However, the information on choline content of many foods from outside North America remains limited, thus making it difficult to accurately estimate dietary choline intake worldwide.

## 4. Dietary Recommendation for Choline

### 4.1. Adequate Intake Recommendation by Stage of the Life Cycle

In 1998, the Food and Nutrition Board of the Institute of Medicine (IOM) published Dietary Reference Intakes (DRIs) for choline (Table 3), as part of a set of reference values for nutrient intakes for healthy populations in the United States (US) and Canada [8]. Due to the lack of sufficient evidence at that time, an Estimated Average Requirement (EARs) for choline could not be calculated; instead, intake recommendations were set as Adequate Intakes (AIs) for total choline. The AI for choline for infants from 0 to 6 months old was set at 125 mg/day, based on a milk volume intake of 0.78 L/day and total choline content of 160 mg/L (1500 µmol/L) [8]. The mean milk volume intake was estimated from test weighing before and after each feeding by healthy, full-term birth infants who were exclusively breastfed [154,155]. It is important to mention that at the time the AI for choline was set, the only one available study, measuring all five individual forms of choline present in mature human milk, reported a lower mean milk concentration of 134 mg/L [121]. However, no rationale was given for the 20% increase in total choline concentration in human milk (134 rounded to 160 mg/L) to establish the AI for early infancy. For infants from 7 to 12 months old, the AI was set at 150 mg/day using a body weight ratio calculation to extrapolate the AI from early infancy. For adults, the AI for choline was set at 550 mg/day for men and 425 mg/day for women. These values were based on the amount (7 mg/kg/day) that prevented hepatic alteration in men, defined as elevated alanine aminotransferase concentration in serum [7], and reference body weights of 76 kg and 59 kg for men and women, respectively [8]. It should be noted that the small depletion-repletion study used to derive these values was conducted only in men and did not provide information on whether less choline would be effective, as researchers only studied one dose [7]. From this, the AIs for children and adolescents were extrapolated using the following formula: AI = AI adult (weight child/weight adult)^0.75^ (1 + growth factor). The growth factors for children are 0.30 between 7 months to 3 years, 0.15 between 4 to 13 years, 0.15 for males between 14 to 18 years, and 0.00 for females between 14 to 18 years [8]. For pregnant women, the AI for choline was set at 450 mg/day for all trimesters, which was calculated as the AI for adult women plus fetal and placental choline accumulation, based on animal data [156,157,158]. For lactating women, the AI for choline was set at 550 mg/day, calculated as the AI for adult women plus an increment to cover choline output in mature milk, using the AI set for early infancy.

The Panel on Dietetics Products, Nutrition, and Allergies from the European Food Safety Authority (EFSA) published the Dietary Reference Values for Choline in 2016 [9]. Similar to IOM, EFSA considered that requirements for choline cannot be estimated, and therefore set AIs for total choline (Table 2). For infants from 7 to 11 months of age, the AI was set at 160 mg/day based on the extrapolation from choline intake of exclusively breastfed infants from 0 to 6 months old (120 mg/day) [9,121,129]. For adults, the AI was set at 400 mg/day based on the mean choline intake from healthy populations observed in the European Union [159,160], and the amount needed to replete most of the depleted subjects with liver/muscle damage [161]. Like the IOM, the AIs for the other stages were extrapolated from the adult value considering growing factors for children, gestational body weight increase for pregnant women, and human milk output for lactating women [9]. Although the majority of the AIs set by the IOM and EFSA are similar, the major difference was for adults; the EFSA set the AI at 400 mg/day for men and women, while the IOM set a different higher AI for men (550 mg/day) in comparison to women (425 mg/day for women). 

### 4.2. Tolerable Upper Intake Levels

The IOM set the UL for choline at 3.5 g/day for adults based on the prevention of hypotension [8]. One study reported a hypotensive effect after the oral administration of 10 g/day of choline chloride (equivalent to 7.5 g choline) [162]. A lowest-observed-adverse-effect level of 7.5 g/day was divided by an uncertainty factor of 2 to obtain a UL of 3.75 g/day for adults, which was rounded down to 3.5 g/day. This value was also used to set a UL for pregnant and lactating women, and the ULs for children and adolescents were extrapolated using body weight. 

In light of the findings of studies conducted after the DRIs were published, it is apparent that the occurrence of fatty liver or muscle damage in individuals consuming choline-deficient diets differs between gender and age groups [161,163]. Specifically, men and postmenopausal women are more susceptible to organ dysfunction compared to premenopausal women, when consuming choline-deficient diets [161,163]. This observation was related to higher estrogen concentrations in premenopausal women, which may enhance the endogenous synthesis of phosphatidylcholine via the PEMT pathway [119]. Recent studies have also identified several single nucleotide polymorphisms that impact PEMT and other enzymes involved in one-carbon metabolism, thus influencing the susceptibility to organ dysfunction [164,165].

## 5. Dietary Assessment Methods and Validation

### 5.1. Dietary Assessment Methods 

Dietary intake can be assessed by different methods, including food records, 24-h recalls (24HRs), and food frequency questionnaires (FFQs). FFQs are widely used to estimate usual dietary intake in large epidemiological surveys [166,167] because of their low participant burden and cost [168]. The purpose of an FFQ is to obtain information on the usual frequency of food consumption and to rank individuals according to their intake level [169]. In some cases, FFQs also include information on portion sizes, allowing the estimation of absolute daily intakes [170]. However, absolute daily intakes from an FFQ are not usually as accurate as those derived from other methods, such as a 24HR or weighed food record, which allow the collection of more detailed information on the portion size of foods consumed [170,171,172,173]. The administration of only one 24HR does not adequately represent the usual intake of an individual, and the number of days required to estimate usual nutrient intakes at the individual level varies considerably, with a range of three to 41 days depending on the nutrient [174]. The numbers of days necessary to assess dietary intake of choline or betaine are not known, and the mean of at least two 24HRs has been suggested for energy intake estimation [175]. Therefore, the method selected to collect dietary intake data can influence the estimation of the nutrient of interest.

An adequate dietary intake assessment of usual choline and betaine intakes, reflecting long-term daily intake, is a prerequisite for the association with health and disease outcomes. The richest sources of total choline are foods of animal origin [2], which may not be consumed on a daily basis. The relationship between low choline, or betaine, intakes and an increased risk of adverse health outcomes has been described in several studies [49,50,51,75,176,177]. However, other studies have found no or only a weak association between choline or betaine intakes and these adverse health outcomes [73,96,178]. The inconsistency of the results that attempt to show the influence of dietary choline and betaine intakes in health outcomes raises the question of the validity of the methods used to assess dietary choline and betaine intakes.

### 5.2. Dietary Assessment Validation 

Valid dietary methods for assessing choline intake are critical to obtaining an estimation of dietary intakes. Given there is often no ‘gold standard’ methodology for assessing dietary nutrient intakes, relative validity is often assessed where a selected method is compared to another reference method [168]. Before using an FFQ, it should be validated in the population and for the nutrient of interest. The mean of multiple 24HRs has frequently been used as a reference method to assess the validity of FFQs [169]. The relative validity is often assessed by the use of different statistical analyses, including correlation coefficients, Bland-Altman plots, cross-classification, and weighted Cohen’s kappa coefficients [179,180,181,182,183,184]. However, only a few recent studies have reported on the validation of methods to assess choline and betaine intakes [159,185,186,187]. This could be related to the fact that the first food composition database for these nutrients only became available for use less than 15 years ago [148]. Moreover, no validation studies assessing the intake of the individual forms of choline have been published to date. 

A tendency for higher absolute choline and betaine intakes estimated from an FFQ compared to the mean intake from the reference method has been reported in the United States and Europe [159,186,187]. In addition, the estimated energy intake tended to be higher in the FFQ compared to 24HR, which supports the overestimation of intakes. A possible explanation for this could be the long list of food items included in FFQs, as participants may lose focus after a while, or the difficulty in assessing portion size or frequency of consumption. Another possibility is that the time frame during which the reference method was administrated was not long enough to adequately quantify the usual intakes of the participants. However, similar time frames have been used in other validation studies [188]. In addition, similar to choline, betaine-rich food sources, such as beets and quinoa [2], may not be frequently consumed and therefore may be captured in the FFQ, but not in the three 24HRs. Based on estimates of within-subject variability for other nutrients, it is probable that additional days of dietary recalls would have been required to improve the accuracy of the 24HRs at the individual level [174,189]. This limitation is inherent in most studies that use 24HRs as the reference method for validation, as reporting accuracy in 24HRs may diminish as the respondent burden increases. It has been recommended to adjust by energy intake before assessing the association of estimated intake values between methods [190]. 

## 6. Dietary Choline Intake by Stage of Life Cycle 

### 6.1. Dietary Choline Intakes in Adults 

Dietary choline intake information is currently available mainly from North American and European countries (Table 4). The first report describing dietary choline intake in adults was published in 2005 in the US [191]. The 2007–2008 National Health and Nutrition Examination Survey (NHANES), which is a population representative sample survey of the US, indicated that the mean choline intake was 396 mg/day for men and 260 mg/day for women using two 24HRs [192]. This report also included information indicating that choline intakes can differ by ethnic background [192]. Comparable estimated mean choline intakes have been reported from 2009–2010, 2011–2012, and 2013–2014 NHANES [193,194]. In Canada, mean total choline intake in adults has been recently estimated at 372 mg/day for men and 292 mg/day for women in Newfoundland using a food frequency questionnaire [195]. 

A recent report describes the dietary choline intake and food sources from national surveys performed in nine European countries [160]. These data showed that the highest dietary intake was collected from people in the Northern countries, whereas Mediterranean countries had the lowest intakes [160]. Other studies reporting dietary choline intake have originated from China [56], Mexico [196], New Zealand [196], and Taiwan [197]. Worldwide, total choline intake in adults ranges from 284 mg/day to 468 mg/day for men, from Taiwan and Sweden, respectively; and from 263 mg/day to 374 mg/day for women, from Mexico and Sweden, respectively. Given that the AIs established by the IOM were set based on a single study conducted in men where only one choline dose was used, it is interesting to note that a common finding is that mean intakes are below the corresponding dietary choline recommendation. The evaluation of choline intake must be done with caution, as intake levels above the AI imply a low probability of inadequate intake, but intake below the AI does not necessarily indicate inadequacy [198]. Therefore, given the definition of AI, no conclusion on the prevalence of choline intake deficiency can be made.

Only a small number of studies have reported on the individual choline forms in addition to total choline intake. In adults, lipid-soluble choline forms contribute between 45 to 60% of total choline intake, with phosphatidylcholine being the major form [199,201,202,203,204,205]. Intakes of water-soluble choline forms (free choline and glycerophosphocholine) contribute approximately 25% and 15% of total choline, respectively [167,185]. The richest food groups identified contributing to dietary choline intake in the US are animal-food sources: Meat, poultry, and fish [192]. Major food sources of dietary choline vary by country. For example, eggs, meat, and dairy are the major sources of total dietary choline in New Zealand [200]. In contrast, eggs, seafood, meats, and soy products are the predominant sources in Japan and China [56,199]. 

### 6.2. Dietary Choline Intakes in Other Age Groups 

In comparison to data obtained from adults in general, there is less information available on dietary choline intake levels related to major food group sources for specific stages of the life cycle, including toddlers, children, adolescents, pregnancy, lactation, and the elderly (Table 5). In North America, the estimated mean choline intake during pregnancy is similar to the choline intake estimated during lactation [54,206]. During pregnancy and lactation, phosphatidylcholine and glycerophosphocholine sources have been described as the main contributors to total choline intake [54,207,208]. As for adults, the estimated mean total choline intakes in pregnant and lactating women are commonly below the recommended AIs [8,9]. A similar situation exists for the elderly [160,194,209]. On the contrary, the mean intake reported for children in Germany is above the current recommended AI [160]. 

Interestingly, no differences have been reported in total choline intake during lactation compared to pregnancy [72,206,210]. This observation is relevant since the AI for choline is higher for lactation compared to pregnancy [8,9]. In humans, higher dietary choline intakes are inversely associated with a risk of neural tube defects [49]. Choline supplementation studies during pregnancy and lactation suggest that maternal choline above the current AI intakes (980 vs. 430 mg/day) decreases preeclampsia risk markers [211] and increases milk choline concentrations [120]. Currently, most of the commercially available prenatal supplements do not contain choline, and the estimated choline intake from supplements has been estimated to be low, ranging between 14 to 25 mg/day [194]. Recently, the American Medical Association adopted the inclusion of choline in all prenatal supplements [212]. However, more research is still required to determine the specific choline requirements for both pregnant and lactating women.

## 7. Summary and Future Directions

Choline is a complex essential nutrient involved in several diverse body functions. It must be obtained from the diet as endogenous synthesis is insufficient to cover the body’s needs. Additionally, choline exists in different water-soluble and lipid-soluble forms in foods; these forms may differ in metabolic fate and subsequent impact on growth and development. Our current knowledge of choline in human milk is limited to relatively few studies, mostly from high-income countries where maternal animal source food consumption is likely higher than that of counterparts from low-income countries. Studies with large sample sizes of healthy lactating women from different countries that assess both total and individual milk choline concentration are needed, as these data may help to generate a potentially more appropriate reference for assessing choline concentrations in human milk. Although the estimations of dietary choline intakes in different studies worldwide are based on the same single database, this source contains information on a limited number of samples per food item and were obtained only from the US. More work is needed to expand the current database as it may not be totally representative of certain foods consumed by a particular population and it is uncertain whether the choline content of foods differs between countries. Is important to mention that only a fraction of the studies on dietary choline intakes are based on data from national surveys; thus, studies with large sample sizes of randomly selected participants are needed. Moreover, direct comparison of the estimates of choline intake from different studies should be done with caution, as different dietary assessment methods used to estimate choline intake may influence the dietary choline intake estimation. At present, there remains limited information available on the usual dietary choline intake across different age groups in the life cycle, particularly for early childhood. Clearly, more research on choline requirements and physiological benefits associated with dietary intake is needed to properly assess the importance of this nutrient. 

## Figures and Tables

**Table 1:** Studies reporting choline concentrations in mature human milk.

Country	*n*	Betaine (µmol/L)	Choline (µmol/L) ^1^						TC (mg/L)	Reference
			FC	PCho	GPC	PC	SM	TC		
US	10	-	85	-	-	180	206	-	-	[127]
US	16	-	116	570	362	82	124	1254	130	[121]
US	48	7	83	553	388	107	67	1198	125	[119]
US	60	-	158	-	-	-	-	-	-	[128]
US	28	3.8	84	500	403	63	175	1225	128	[120]
Turkey	12	-	286	438	465	155	97	1441 ^2^	150	[116]
Turkey ^3^	54	-	93	351	958	-	-	1532	159	[129]
Japan	62	-	-	-	-	-	-	950	99	[117]
Korea	36	31	283	-	-	-	-	1600	166	[118]
Sweden	1	-	188	704	672	-	-	-	-	[130]
Canada	301	4.8	155	535	416	-	-	-	-	[131]
Cambodia	67	5.1	143	562	390	-	-	-	-	[131]

^1^ Data are presented as mean concentrations, unless otherwise noted; ^2^ TC is reported as the sum of all individual choline forms when a discrepancy was found with the value reported for TC in each study; ^3^ median is presented. Abbreviations: FC, free choline; PCho, phosphocholine; GPC, glycerophosphocholine; PC, phosphatidylcholine; SM, sphingomyelin; TC, total choline (sum FC + PCho + GPC + PC + SM).

**Table 2:** Choline and betaine content in different food sources (mg per 100 g of weight).

Food Item	Betaine	Choline					
		FC	PCho	GPC	PC	SM	TC
Beef liver, cooked	5.6	62.0	12.0	83.0	250.0	24.0	431.0 ^1^
Egg, hard boiled	0.6	0.7	0.5	0.5	210.0	14.0	225.7 ^1^
Beef steak, cooked	13.0	0.7	1.3	5.2	86.0	11.0	104.2 ^1^
Salmon, cooked	1.8	7.8	1.2	41.0	37.0	3.4	90.4 ^1^
Pork chops, cooked	2.8	1.1	0.6	12.0	57.0	7.5	78.2
Chicken breast, cooked	6.4	3.2	2.1	1.6	46.0	8.9	61.8
Nuts, almonds	0.5	9.4	1.9	1.2	40.0	0.0	52.5 ^1^
Broccoli, cooked	0.1	8.5	9.3	1.3	21.0	0.0	40.1
Beans, baked canned	0.1	17.0	0.8	1.3	12.0	0.0	31.1 ^1^
Milk, 2% fat	0.9	2.8	1.6	10.0	1.2	0.9	16.5 ^1^
Red potato, cooked	0.2	8.5	1.2	3.8	5.3	0.0	18.8
White rice, cooked	0.3	0.7	0.0	1.0	0.4	0.0	2.1

^1^ TC is reported as the sum of all individual choline forms when a discrepancy was found with the value reported for TC in the database. Abbreviations: FC, free choline; PCho, phosphocholine; GPC, glycerophosphocholine; PC, phosphatidylcholine; SM, sphingomyelin; TC, total choline (sum FC + PCho + GPC + PC + SM). Source: USDA choline content database release two [2].

**Table 3:** Current adequate intake recommendations for choline.

Stage	IOM—1998 ^1^				EFSA—2016 ^2^	
	Age	AI (mg/day)		UL (mg/day)	Age	AI (mg/day)
		Males	Females			
Infants	0–6 month	125	125	-	0–6 month	120
	7–12 month	150	150	-	7–11 month	160
Children	1–3 year	200	200	1000	1–3 year	140
	4–8 year	250	250	1000	4–6 year	170
	9–13 year	375	375	2000	7–10 year	250
	14–18 year	550	400	3000	11–14 year	340
					15–17 year	400
Adults	≥19 year	550	425	3500	≥18 year	400
Pregnancy	-	-	450	3000	-	480
Lactation	-	-	550	3500	-	520

^1^ Dietary Reference Intakes for choline from the Institute of Medicine (IOM) [8]; ^2^ Dietary Reference Values for choline from the European Food Safety Authority (EFSA) [9].

**Table 4:** Studies reporting on dietary total choline intake in healthy adults.

Country	Dietary Method	Total Choline Intake (mg/day) ^1^				Reference
		Men		Women		
		*n*	Intake	*n*	Intake	
Canada	FFQ	822	372 ± 287	2232	292 ± 213	[195]
China	FFQ	18,763	318 ± 92	37,432	289 ± 85	[56]
Finland ^4^	48HR ×2	585	450 (425) ^2^	710	344 (327) ^2^	[160]
France ^4^	DR ×7	936	370 (362) ^2^	1340	291 (283) ^2^	[160]
Greece	FFQ	1514	291 ± 79	1528	285 ± 75	[50]
Japan	FFQ	13,355	445–513 ^3^	15,724	388–442 ^3^	[199]
Italy ^4^	DR ×3	1068	357 (341) ^2^	1245	293 (282) ^2^	[160]
Ireland ^4^	DR ×4	634	461 (443) ^2^	640	318 (314) ^2^	[160]
Mexico	FFQ	-	-	1027	263 ± 105	[196]
New Zealand	WFR ×3	-	-	125	316 ± 65	[200]
Sweden ^4^	DR ×4	623	468 (442) ^2^	807	374 (356) ^2^	[160]
Taiwan	FFQ	321	284 ± 145	227	230 ± 120	[197]
The Netherlands ^4^	24HR ×2	1023	448 (425) ^2^	1034	334 (317) ^2^	[160]
UK ^4^	DR ×4	560	407 (385) ^2^	706	294 (282) ^2^	[160]
US ^4^	24HR ×2	2563	421 (408) ^2^	2704	279 (271) ^2^	[194]

^1^ Data are presented as mean ± SD, unless otherwise noted; ^2^ mean (median); ^3^ interquartile range; ^4^ data were obtained from national representative surveys. Abbreviations: 24HR, 24-h recall; 48HR, 48-h recall; DR, dietary record; FFQ, food frequency questionnaire; WFR, weighted food record. ×1–×7 = number of days collected.

**Table 5:** Studies reporting on dietary choline intake in other age groups.

Age Group and Country	Dietary Method	Subgroup	Total Choline Intake (mg/day) ^1^		Reference
			*n*	Intake	
Toddlers (1–3 year)					
Canada	DR ×3	1 year	110	174 ± 56	[74]
Finland ^5^	DR ×3	1–3 year	500	176 (172) ^2,4^	[160]
US ^5^	24HR ×2	2–3 year	1316	224 (217) ^2^	[194]
Children (4–9 year)					
Canada	FFQ	6 year	193	302 ± 100	[213]
Germany ^5^	DR ×3	6–10 year	835	288 (276) ^2,4^	[160]
Romania	DR ×3	4–6 year	71	215 ± 32	[214]
US ^5^	24HR ×2	4–8 year	2774	243 (235) ^2^	[194]
Adolescents (10–18 year)					
Bangladesh	FFQ	Boys and girls	47	128 ± 3.2 ^3^	[215]
The Netherlands	24HR ×2	10–18 year, boys	566	353 (338) ^2^	[160]
		10–18 year, girls	576	291 (279) ^2^	
US ^5^	24HR ×2	14–18 year, boys	1207	295 (288) ^2^	[194]
		14–18 year, girls	1147	244 (237) ^2^	
Pregnancy					
Bangladesh	24HR ×1	T3	103	190 ± 98	[216]
Belgium	FFQ	T2	85	268 ± 7.4 ^3^	[210]
Canada	FFQ	T3	290	302 ± 122	[208]
Jamaica	FFQ	T1	16	279 ± 116	[217]
Latvia	24HR ×2	T1–T3	990	356 (330) ^2^	[160]
US ^5^	24HR ×2	T1–T3	593	319 (309) ^2^	[194]
Lactation					
Belgium	FFQ	6 mpp	60	268 ± 7.8 ^3^	[210]
Canada	24HR ×1	3 mpp	488	346 ± 151	[206]
US	DR ×3	1.5 mpp	98	356 ± 109 ^4^	[72]
Elderly (> 65 year)					
Italy ^5^	DR ×3	Men	69	335 (320) ^2^	[160]
		Women	159	269 (269) ^2^	
Poland	FFQ	Women	122	392 ± 26 ^3^	[209]
US ^5^	24HR ×2	Men	1099	363 (351) ^2^	[194]
		Women	1145	266 (259) ^2^	

^1^ Data are presented as mean ± SD, unless otherwise noted; ^2^ mean (median); ^3^ mean ± SE; ^4^ mean data are presented; ^5^ data were obtained from national representative surveys. Abbreviations: 24HR, 24-h recall; DR, dietary record; FFQ, food frequency questionnaire; mpp, months postpartum; T1, first trimester; T2, second trimester; T3, third trimester. ×1–×3 = number of days collected.

## References

[1] Vance D.E., Ridgway N.D. (1988). The methylation of phosphatidylethanolamine. Prog. Lipid Res..

[2] Patterson Y.K., Bhagwat A.S., Williams R.J., Howe C.J., Holden M.J. (2008). USD Database for The Choline Content of Common Foods, Release 2.

[3] Zeisel S.H. (2006). The fetal origins of memory: The role of dietary choline in optimal brain development. J Pediatr..

[4] Zeisel S.H. (2006). Choline: Critical role during fetal development and dietary requirements in adults. Annu. Rev. Nutr..

[5] Leermakers E.T., Moreira E.M., Kiefte-de J.C., Darweesh S.K., Visser T., Voortman T., Bautista P.K., Chowdhury R., Gorman D., Bramer W.M. (2015). Effects of choline on health across the life course: A systematic review. Nutr. Rev..

[6] Buchman A.L., Moukarzel A., Jenden D.J., Roch M., Rice K., Ament M.E. (1993). Low plasma free choline is prevalent in patients receiving long term parenteral nutrition and is associated with hepatic aminotransferase abnormalities. Clin. Nutr..

[7] Zeisel S.H., Da Costa K.A., Franklin P.D., Alexander E.A., Lamont J.T., Sheard N.F., Beiser A. (1991). Choline, an essential nutrient for humans. FASEB J..

[8] Institute of Medicine (1998). Dietary Reference Intakes for Thiamin, Riboflavin, Niacin, Vitamin B6, Folate, Vitamin B12, Pantothenic Acid, Biotin, and Choline.

[9] European Food Safety Authority (2016). Dietary reference values for choline. EFSA J..

[10] Zeisel S.H. (1981). Dietary choline: Biochemistry, physiology, and pharmacology. Annu. Rev. Nutr..

[11] Lewis E.D., Field C.J., Jacobs R.L. (2015). Should the forms of dietary choline also be considered when estimating dietary intake and the implications for health?. Lipid Technol..

[12] Cheng W.-L., Holmes-McNary M.Q., Mar M.-H., Lien E.L., Zeisel S.H. (1996). Bioavailability of choline and choline esters from milk in rat pups. J. Nutr. Biochem..

[13] Lewis E.D., Richard C., Goruk S., Dellschaft N.S., Curtis J.M., Jacobs R.L., Field C.J. (2016). The form of choline in the maternal diet affects immune development in suckled rat offspring. J. Nutr..

[14] Richard C., Lewis E.D., Goruk S., Wadge E., Curtis J.M., Jacobs R.L., Field C.J. (2017). Feeding a mixture of choline forms to lactating dams improves the development of the immune system in sprague-dawley rat offspring. Nutrients.

[15] Dellschaft N.S., Richard C., Lewis E.D., Goruk S., Jacobs R.L., Curtis J.M., Field C.J. (2018). The dietary form of choline during lactation affects maternal immune function in rats. Eur. J. Nutr..

[16] Sarter M., Parikh V. (2005). Choline transporters, cholinergic transmission and cognition. Nat. Rev. Neurosci..

[17] Varoqui H., Erickson J.D. (1998). The cytoplasmic tail of the vesicular acetylcholine transporter contains a synaptic vesicle targeting signal. J. Biol. Chem..

[18] Kawashima K., Fujii T. (2008). Basic and clinical aspects of non-neuronal acetylcholine: Overview of non-neuronal cholinergic systems and their biological significance. J. Pharmacol. Sci..

[19] Wessler I., Kirkpatrick C.J. (2008). Acetylcholine beyond neurons: The non-neuronal cholinergic system in humans. Br. J. Pharmacol..

[20] Zeisel S.H., Wishnok J.S., Blusztajn J.K. (1983). Formation of methylamines from ingested choline and lecithin. J. Pharmacol. Exp. Ther..

[21] Romano K.A., Vivas E.I., Amador-Noguez D., Rey F.E. (2015). Intestinal microbiota composition modulates choline bioavailability from diet and accumulation of the proatherogenic metabolite trimethylamine-*N*-oxide. MBio.

[22] Baker J.R., Chaykin S. (1962). The biosynthesis of trimethylamine-*N*-oxide. J. Biol. Chem..

[23] Bianchi G., Azzone G.F. (1964). Oxidation of choline in rat liver mitochondria. J. Biol. Chem..

[24] Munoz-Clares R.A., Diaz-Sanchez A.G., Gonzalez-Segura L., Montiel C. (2010). Kinetic and structural features of betaine aldehyde dehydrogenases: Mechanistic and regulatory implications. Arch. Biochem. Biophys..

[25] Garrow T.A. (1996). Purification, kinetic properties, and cdna cloning of mammalian betaine-homocysteine methyltransferase. J. Biol. Chem..

[26] Delgado-Reyes C.V., Wallig M.A., Garrow T.A. (2001). Immunohistochemical detection of betaine-homocysteine S-methyltransferase in human, pig, and rat liver and kidney. Arch. Biochem. Biophys..

[27] Lever M., Slow S. (2010). The clinical significance of betaine, an osmolyte with a key role in methyl group metabolism. Clin. Biochem..

[28] Feng Q., Kalari K., Fridley B.L., Jenkins G., Ji Y., Abo R., Hebbring S., Zhang J., Nye M.D., Leeder J.S. (2011). Betaine-homocysteine methyltransferase: Human liver genotype-phenotype correlation. Mol. Genet. Metab..

[29] Pajares M.A., Perez-Sala D. (2006). Betaine homocysteine S-methyltransferase: Just a regulator of homocysteine metabolism?. Cell Mol. Life Sci..

[30] Zeisel S.H. (2012). Dietary choline deficiency causes DNA strand breaks and alters epigenetic marks on DNA and histones. Mutat. Res..

[31] Haubrich D.R., Gerber N.H. (1981). Choline dehydrogenase. Assay, properties and inhibitors. Biochem. Pharmacol..

[32] Grossman E.B., Hebert S.C. (1989). Renal inner medullary choline dehydrogenase activity: Characterization and modulation. Am. J. Physiol..

[33] McKeever M.P., Weir D.G., Molloy A., Scott J.M. (1991). Betaine-homocysteine methyltransferase: Organ distribution in man, pig and rat and subcellular distribution in the rat. Clin. Sci. (Lond.).

[34] Soloway S., Stetten D. (1953). The metabolism of choline and its conversion to glycine in the rat. J. Biol. Chem..

[35] Li Z., Vance D.E. (2008). Phosphatidylcholine and choline homeostasis. J. Lipid Res..

[36] Kennedy E.P. (1956). The synthesis of cytidine diphosphate choline, cytidine diphosphate ethanolamine, and related compounds. J. Biol. Chem..

[37] Sundler R., Akesson B. (1975). Biosynthesis of phosphatidylethanolamines and phosphatidylcholines from ethanolamine and choline in rat liver. Biochem. J..

[38] DeLong C.J., Shen Y.J., Thomas M.J., Cui Z. (1999). Molecular distinction of phosphatidylcholine synthesis between the CDP-choline pathway and phosphatidylethanolamine methylation pathway. J. Biol. Chem..

[39] Bremer J., Greenberg D.M. (1960). Biosynthesis of choline in vitro. Biochim. Biophys. Acta.

[40] Vance J.E. (2008). Phosphatidylserine and phosphatidylethanolamine in mammalian cells: Two metabolically related aminophospholipids. J. Lipid Res..

[41] Ridgway N.D., Yao Z., Vance D.E. (1989). Phosphatidylethanolamine levels and regulation of phosphatidylethanolamine *N*-methyltransferase. J. Biol. Chem..

[42] Cantoni G.L. (1975). Biological methylation: Selected aspects. Annu. Rev. Biochem..

[43] Infante J.P., Kinsella J.E. (1976). Phospholipid synthesis in mammary tissue. Choline and ethanolamine kinases: Kinetic evidence for two discrete active sites. Lipids.

[44] Bjornstad P. (1966). Phospholipase activity in rat liver mitochondria studied by the use of endogenous substrates. J. Lipid Res..

[45] Noga A.A., Vance D.E. (2003). Insights into the requirement of phosphatidylcholine synthesis for liver function in mice. J. Lipid Res..

[46] Horl G., Wagner A., Cole L.K., Malli R., Reicher H., Kotzbeck P., Kofeler H., Hofler G., Frank S., Bogner-Strauss J.G. (2011). Sequential synthesis and methylation of phosphatidylethanolamine promote lipid droplet biosynthesis and stability in tissue culture and in vivo. J. Biol. Chem..

[47] Da Costa K.A., Sanders L.M., Fischer L.M., Zeisel S.H. (2011). Docosahexaenoic acid in plasma phosphatidylcholine may be a potential marker for in vivo phosphatidylethanolamine *N*-methyltransferase activity in humans. Am. J. Clin. Nutr..

[48] West A.A., Yan J., Jiang X., Perry C.A., Innis S.M., Caudill M.A. (2013). Choline intake influences phosphatidylcholine DHA enrichment in nonpregnant women but not in pregnant women in the third trimester. Am. J. Clin. Nutr..

[49] Shaw G.M., Carmichael S.L., Yang W., Selvin S., Schaffer D.M. (2004). Periconceptional dietary intake of choline and betaine and neural tube defects in offspring. Am. J. Epidemiol..

[50] Detopoulou P., Panagiotakos D.B., Antonopoulou S., Pitsavos C., Stefanadis C. (2008). Dietary choline and betaine intakes in relation to concentrations of inflammatory markers in healthy adults: The ATTICA study. Am. J. Clin. Nutr..

[51] Xu X., Gammon M.D., Zeisel S.H., Lee Y.L., Wetmur J.G., Teitelbaum S.L., Bradshaw P.T., Neugut A.I., Santella R.M., Chen J. (2008). Choline metabolism and risk of breast cancer in a population-based study. FASEB J..

[52] Shaw G.M., Finnell R.H., Blom H.J., Carmichael S.L., Vollset S.E., Yang W., Ueland P.M. (2009). Choline and risk of neural tube defects in a folate-fortified population. Epidemiology.

[53] Wu B.T., Dyer R.A., King D.J., Richardson K.J., Innis S.M. (2012). Early second trimester maternal plasma choline and betaine are related to measures of early cognitive development in term infants. PLoS ONE.

[54] Boeke C.E., Gillman M.W., Hughes M.D., Rifas-Shiman S.L., Villamor E., Oken E. (2013). Choline intake during pregnancy and child cognition at age 7 years. Am. J. Epidemiol..

[55] Strain J.J., McSorley E.M., van Wijngaarden E., Kobrosly R.W., Bonham M.P., Mulhern M.S., McAfee A.J., Davidson P.W., Shamlaye C.F., Henderson J. (2013). Choline status and neurodevelopmental outcomes at 5 years of age in the Seychelles child development nutrition study. Br. J. Nutr..

[56] Yu D., Shu X.O., Xiang Y.B., Li H., Yang G., Gao Y.T., Zheng W., Zhang X. (2014). Higher dietary choline intake is associated with lower risk of nonalcoholic fatty liver in normal-weight chinese women. J. Nutr..

[57] Sun S., Li X., Ren A., Du M., Du H., Shu Y., Zhu L., Wang W. (2016). Choline and betaine consumption lowers cancer risk: A meta-analysis of epidemiologic studies. Sci. Rep..

[58] Zhou R.F., Chen X.L., Zhou Z.G., Zhang Y.J., Lan Q.Y., Liao G.C., Chen Y.M., Zhu H.L. (2017). Higher dietary intakes of choline and betaine are associated with a lower risk of primary liver cancer: A case-control study. Sci. Rep..

[59] Obeid R., Awwad H.M., Knell A.I., Hubner U., Geisel J. (2018). Glucose and fat tolerance tests induce differential responses in plasma choline metabolites in healthy subjects. Nutrients.

[60] Rees W.D., Hay S.M., Cruickshank M. (2006). An imbalance in the methionine content of the maternal diet reduces postnatal growth in the rat. Metabolism.

[61] Luka Z., Mudd S.H., Wagner C. (2009). Glycine *N*-methyltransferase and regulation of S-adenosylmethionine levels. J. Biol. Chem..

[62] Niculescu M.D., Craciunescu C.N., Zeisel S.H. (2006). Dietary choline deficiency alters global and gene-specific DNA methylation in the developing hippocampus of mouse fetal brains. FASEB J..

[63] Kovacheva V.P., Mellott T.J., Davison J.M., Wagner N., Lopez-Coviella I., Schnitzler A.C., Blusztajn J.K. (2007). Gestational choline deficiency causes global and igf2 gene DNA hypermethylation by up-regulation of dnmt1 expression. J. Biol. Chem..

[64] Davison J.M., Mellott T.J., Kovacheva V.P., Blusztajn J.K. (2009). Gestational choline supply regulates methylation of histone h3, expression of histone methyltransferases g9a (kmt1c) and suv39h1 (kmt1a), and DNA methylation of their genes in rat fetal liver and brain. J. Biol. Chem..

[65] Jiang X., Yan J., West A.A., Perry C.A., Malysheva O.V., Devapatla S., Pressman E., Vermeylen F., Caudill M.A. (2012). Maternal choline intake alters the epigenetic state of fetal cortisol-regulating genes in humans. FASEB J..

[66] Shaw G.M., Carmichael S.L., Laurent C., Rasmussen S.A. (2006). Maternal nutrient intakes and risk of orofacial clefts. Epidemiology.

[67] Yang W., Shaw G.M., Carmichael S.L., Rasmussen S.A., Waller D.K., Pober B.R., Anderka M., National Birth Defects Prevention Study (2008). Nutrient intakes in women and congenital diaphragmatic hernia in their offspring. Birth Defects Res. A Clin. Mol. Teratol..

[68] Carmichael S.L., Yang W., Correa A., Olney R.S., Shaw G.M., National Birth Defects Prevention Study (2009). Hypospadias and intake of nutrients related to one-carbon metabolism. J. Urol..

[69] Chan J., Deng L., Mikael L.G., Yan J., Pickell L., Wu Q., Caudill M.A., Rozen R. (2010). Low dietary choline and low dietary riboflavin during pregnancy influence reproductive outcomes and heart development in mice. Am. J. Clin. Nutr..

[70] Meck W.H., Williams C.L. (2003). Metabolic imprinting of choline by its availability during gestation: Implications for memory and attentional processing across the lifespan. Neurosci. Biobehav. Rev..

[71] Signore C., Ueland P.M., Troendle J., Mills J.L. (2008). Choline concentrations in human maternal and cord blood and intelligence at 5 y of age. Am. J. Clin. Nutr..

[72] Cheatham C.L., Goldman B.D., Fischer L.M., da Costa K.A., Reznick J.S., Zeisel S.H. (2012). Phosphatidylcholine supplementation in pregnant women consuming moderate-choline diets does not enhance infant cognitive function: A randomized, double-blind, placebo-controlled trial. Am. J. Clin. Nutr..

[73] Villamor E., Rifas-Shiman S.L., Gillman M.W., Oken E. (2012). Maternal intake of methyl-donor nutrients and child cognition at 3 years of age. Paediatr. Perinat. Epidemiol..

[74] Wiedeman A.M., Chau C.M.Y., Grunau R.E., McCarthy D., Yurko-Mauro K., Dyer R.A., Innis S.M., Devlin A.M. (2018). Plasma betaine is positively associated with developmental outcomes in healthy toddlers at age 2 years who are not meeting the recommended adequate intake for dietary choline. J. Nutr..

[75] Poly C., Massaro J.M., Seshadri S., Wolf P.A., Cho E., Krall E., Jacques P.F., Au R. (2011). The relation of dietary choline to cognitive performance and white-matter hyperintensity in the Framingham offspring cohort. Am. J. Clin. Nutr..

[76] Nurk E., Refsum H., Bjelland I., Drevon C.A., Tell G.S., Ueland P.M., Vollset S.E., Engedal K., Nygaard H.A., Smith D.A. (2013). Plasma free choline, betaine and cognitive performance: The Hordaland health study. Br. J. Nutr..

[77] Ladd S.L., Sommer S.A., LaBerge S., Toscano W. (1993). Effect of phosphatidylcholine on explicit memory. Clin. Neuropharmacol..

[78] Spiers P.A., Myers D., Hochanadel G.S., Lieberman H.R., Wurtman R.J. (1996). Citicoline improves verbal memory in aging. Arch. Neurol..

[79] Benton D., Donohoe R.T. (2004). The influence on cognition of the interactions between lecithin, carnitine and carbohydrate. Psychopharmacology (Berl.).

[80] Knott V., de la Salle S., Choueiry J., Impey D., Smith D., Smith M., Beaudry E., Saghir S., Ilivitsky V., Labelle A. (2015). Neurocognitive effects of acute choline supplementation in low, medium and high performer healthy volunteers. Pharmacol. Biochem. Behav..

[81] Naber M., Hommel B., Colzato L.S. (2015). Improved human visuomotor performance and pupil constriction after choline supplementation in a placebo-controlled double-blind study. Sci. Rep..

[82] Lippelt D.P., van der Kint S., van Herk K., Naber M. (2016). No acute effects of choline bitartrate food supplements on memory in healthy, young, human adults. PLoS ONE.

[83] Da Costa K.A., Badea M., Fischer L.M., Zeisel S.H. (2004). Elevated serum creatine phosphokinase in choline-deficient humans: Mechanistic studies in C_2_C_12_ mouse myoblasts. Am. J. Clin. Nutr..

[84] Wirtz K.W., Zilversmit D.B. (1968). Exchange of phospholipids between liver mitochondria and microsomes in vitro. J. Biol. Chem..

[85] Devaux P.F. (1991). Static and dynamic lipid asymmetry in cell membranes. Biochemistry.

[86] Li Z., Agellon L.B., Allen T.M., Umeda M., Jewell L., Mason A., Vance D.E. (2006). The ratio of phosphatidylcholine to phosphatidylethanolamine influences membrane integrity and steatohepatitis. Cell Metab..

[87] Ling J., Chaba T., Zhu L.F., Jacobs R.L., Vance D.E. (2012). Hepatic ratio of phosphatidylcholine to phosphatidylethanolamine predicts survival after partial hepatectomy in mice. Hepatology.

[88] Higgins J.A., Fieldsend J.K. (1987). Phosphatidylcholine synthesis for incorporation into membranes or for secretion as plasma lipoproteins by golgi membranes of rat liver. J. Lipid Res..

[89] Vance D.E. (2008). Role of phosphatidylcholine biosynthesis in the regulation of lipoprotein homeostasis. Curr. Opin. Lipidol..

[90] Corbin K.D., Zeisel S.H. (2012). Choline metabolism provides novel insights into nonalcoholic fatty liver disease and its progression. Curr. Opin. Gastroenterol..

[91] Guerrerio A.L., Colvin R.M., Schwartz A.K., Molleston J.P., Murray K.F., Diehl A., Mohan P., Schwimmer J.B., Lavine J.E., Torbenson M.S. (2012). Choline intake in a large cohort of patients with nonalcoholic fatty liver disease. Am. J. Clin. Nutr..

[92] Zeisel S.H. (1992). Choline: An important nutrient in brain development, liver function and carcinogenesis. J. Am. Coll. Nutr..

[93] Buchman A.L., Ament M.E., Sohel M., Dubin M., Jenden D.J., Roch M., Pownall H., Farley W., Awal M., Ahn C. (2001). Choline deficiency causes reversible hepatic abnormalities in patients receiving parenteral nutrition: Proof of a human choline requirement: A placebo-controlled trial. J. Parenter. Enteral. Nutr..

[94] Gerhard G.T., Duell P.B. (1999). Homocysteine and atherosclerosis. Curr. Opin. Lipidol..

[95] Leach N.V., Dronca E., Vesa S.C., Sampelean D.P., Craciun E.C., Lupsor M., Crisan D., Tarau R., Rusu R., Para I. (2014). Serum homocysteine levels, oxidative stress and cardiovascular risk in non-alcoholic steatohepatitis. Eur. J. Intern. Med..

[96] Dalmeijer G.W., Olthof M.R., Verhoef P., Bots M.L., van der Schouw Y.T. (2008). Prospective study on dietary intakes of folate, betaine, and choline and cardiovascular disease risk in women. Eur. J. Clin. Nutr..

[97] Konstantinova S.V., Tell G.S., Vollset S.E., Nygard O., Bleie O., Ueland P.M. (2008). Divergent associations of plasma choline and betaine with components of metabolic syndrome in middle age and elderly men and women. J. Nutr..

[98] Meyer K.A., Shea J.W. (2017). Dietary choline and betaine and risk of CVD: A systematic review and meta-analysis of prospective studies. Nutrients.

[99] Schwab U., Torronen A., Toppinen L., Alfthan G., Saarinen M., Aro A., Uusitupa M. (2002). Betaine supplementation decreases plasma homocysteine concentrations but does not affect body weight, body composition, or resting energy expenditure in human subjects. Am. J. Clin. Nutr..

[100] Steenge G.R., Verhoef P., Katan M.B. (2003). Betaine supplementation lowers plasma homocysteine in healthy men and women. J. Nutr..

[101] Olthof M.R., Brink E.J., Katan M.B., Verhoef P. (2005). Choline supplemented as phosphatidylcholine decreases fasting and postmethionine-loading plasma homocysteine concentrations in healthy men. Am. J. Clin. Nutr..

[102] Huang T., Chen Y., Yang B., Yang J., Wahlqvist M.L., Li D. (2012). Meta-analysis of B vitamin supplementation on plasma homocysteine, cardiovascular and all-cause mortality. Clin. Nutr..

[103] Pan Y., Guo L.L., Cai L.L., Zhu X.J., Shu J.L., Liu X.L., Jin H.M. (2012). Homocysteine-lowering therapy does not lead to reduction in cardiovascular outcomes in chronic kidney disease patients: A meta-analysis of randomised, controlled trials. Br. J. Nutr..

[104] Tang W.H., Wang Z., Levison B.S., Koeth R.A., Britt E.B., Fu X., Wu Y., Hazen S.L. (2013). Intestinal microbial metabolism of phosphatidylcholine and cardiovascular risk. N. Engl. J. Med..

[105] Mente A., Chalcraft K., Ak H., Davis A.D., Lonn E., Miller R., Potter M.A., Yusuf S., Anand S.S., McQueen M.J. (2015). The relationship between trimethylamine-*N*-oxide and prevalent cardiovascular disease in a multiethnic population living in Canada. Can. J. Cardiol..

[106] Zhu W., Gregory J.C., Org E., Buffa J.A., Gupta N., Wang Z., Li L., Fu X., Wu Y., Mehrabian M. (2016). Gut microbial metabolite TMAO enhances platelet hyperreactivity and thrombosis risk. Cell.

[107] Miller C.A., Corbin K.D., da Costa K.A., Zhang S., Zhao X., Galanko J.A., Blevins T., Bennett B.J., O’Connor A., Zeisel S.H. (2014). Effect of egg ingestion on trimethylamine-*N*-oxide production in humans: A randomized, controlled, dose-response study. Am. J. Clin. Nutr..

[108] DiMarco D.M., Missimer A., Murillo A.G., Lemos B.S., Malysheva O.V., Caudill M.A., Blesso C.N., Fernandez M.L. (2017). Intake of up to 3 eggs/day increases hdl cholesterol and plasma choline while plasma trimethylamine-*N*-oxide is unchanged in a healthy population. Lipids.

[109] Mueller D.M., Allenspach M., Othman A., Saely C.H., Muendlein A., Vonbank A., Drexel H., von Eckardstein A. (2015). Plasma levels of trimethylamine-*N*-oxide are confounded by impaired kidney function and poor metabolic control. Atherosclerosis.

[110] Romano K.A., Martinez-Del C.A., Kasahara K., Chittim C.L., Vivas E.I., Amador-Noguez D., Balskus E.P., Rey F.E. (2017). Metabolic, epigenetic, and transgenerational effects of gut bacterial choline consumption. Cell Host Microbe.

[111] Landfald B., Valeur J., Berstad A., Raa J. (2017). Microbial trimethylamine-*N*-oxide as a disease marker: Something fishy?. Microb. Ecol. Health Dis..

[112] Kruger R., Merz B., Rist M.J., Ferrario P.G., Bub A., Kulling S.E., Watzl B. (2017). Associations of current diet with plasma and urine TMAO in the KarMeN study: Direct and indirect contributions. Mol. Nutr. Food Res..

[113] World Health Organization (2001). The World Health Organization’s Infant Feeding Recommendation.

[114] Zeisel S.H., Wurtman R.J. (1981). Developmental changes in rat blood choline concentration. Biochem. J..

[115] Holmes H.C., Snodgrass G.J., Iles R.A. (2000). Changes in the choline content of human breast milk in the first 3 weeks after birth. Eur. J. Pediatr..

[116] Ilcol Y.O., Ozbek R., Hamurtekin E., Ulus I.H. (2005). Choline status in newborns, infants, children, breast-feeding women, breast-fed infants and human breast milk. J. Nutr. Biochem..

[117] Sakurai T., Furukawa M., Asoh M., Kanno T., Kojima T., Yonekubo A. (2005). Fat-soluble and water-soluble vitamin contents of breast milk from Japanese women. J. Nutr. Sci. Vitaminol. (Tokyo).

[118] Hanok J.Y.S., Chung Y.-J. (2010). Choline and betaine concentrations in breast milk of Korean lactating women and the choline and betaine intakes of their infants. Korean J. Nutr..

[119] Fischer L.M., da Costa K.A., Galanko J., Sha W., Stephenson B., Vick J., Zeisel S.H. (2010). Choline intake and genetic polymorphisms influence choline metabolite concentrations in human breast milk and plasma. Am. J. Clin. Nutr..

[120] Davenport C., Yan J., Taesuwan S., Shields K., West A.A., Jiang X., Perry C.A., Malysheva O.V., Stabler S.P., Allen R.H. (2015). Choline intakes exceeding recommendations during human lactation improve breast milk choline content by increasing pemt pathway metabolites. J. Nutr. Biochem..

[121] Holmes-McNary M.Q., Cheng W.L., Mar M.H., Fussell S., Zeisel S.H. (1996). Choline and choline esters in human and rat milk and in infant formulas. Am. J. Clin. Nutr..

[122] Bitman J., Wood D.L., Mehta N.R., Hamosh P., Hamosh M. (1984). Comparison of the phospholipid composition of breast milk from mothers of term and preterm infants during lactation. Am. J. Clin. Nutr..

[123] Patton S., Keenan T.W. (1975). The milk fat globule membrane. Biochim. Biophys. Acta..

[124] Chao C.K., Pomfret E.A., Zeisel S.H. (1988). Uptake of choline by rat mammary-gland epithelial cells. Biochem. J..

[125] Yang E.K., Blusztajn J.K., Pomfret E.A., Zeisel S.H. (1988). Rat and human mammary tissue can synthesize choline moiety via the methylation of phosphatidylethanolamine. Biochem. J..

[126] Holmes H.C., Snodgrass G.J., Iles R.A. (1996). The choline content of human breast milk expressed during the first few weeks of lactation. Biochem. Soc. Trans..

[127] Zeisel S.H., Char D., Sheard N.F. (1986). Choline, phosphatidylcholine and sphingomyelin in human and bovine milk and infant formulas. J. Nutr..

[128] Cheatham C.L., Sheppard K.W. (2015). Synergistic effects of human milk nutrients in the support of infant recognition memory: An observational study. Nutrients.

[129] Ozarda Y., Cansev M., Ulus I.H. (2014). Breast milk choline contents are associated with inflammatory status of breastfeeding women. J. Hum. Lact..

[130] Wu J., Domellof M., Zivkovic A.M., Larsson G., Ohman A., Nording M.L. (2016). NMR-based metabolite profiling of human milk: A pilot study of methods for investigating compositional changes during lactation. Biochem. Biophys. Res. Commun..

[131] Strecker A. (1842). Notiz über die zusammensetzung des leucins. Liebigs Ann. Chem..

[132] Allen L.H. (2012). B vitamins in breast milk: Relative importance of maternal status and intake, and effects on infant status and function. Adv. Nutr..

[133] Zeisel S.H., Stanbury J.B., Wurtman R.J., Brigida M., Fierro-Benitez R. (1982). Choline content of mothers’ milk in Ecuador and Boston. N. Engl. J. Med..

[134] Food and Agriculture Organization of the United Nations, Statistical Division (2013). FAOSTAT Statistics Database. www.fao.org/faostat/en/#compare.

[135] McDonald C.M., McLean J., Kroeun H., Talukder A., Lynd L.D., Green T.J. (2015). Household food insecurity and dietary diversity as correlates of maternal and child undernutrition in rural Cambodia. Eur. J. Clin. Nutr..

[136] Mark H.E., Houghton L.A., Gibson R.S., Monterrosa E., Kraemer K. (2016). Estimating dietary micronutrient supply and the prevalence of inadequate intakes from national food balance sheets in the South Asia regiona. Asia Pac. J. Clin. Nutr..

[137] Wiedeman A.M., Whitfield K.C., March K.M., Chen N.N., Kroeun H., Sokhoing L., Sophonneary P., Dyer R.A., Xu Z., Kitts D.D. (2018). Concentrations of water-soluble forms of choline in human milk from lactating women in Canada and Cambodia. Nutrients.

[138] Miller E.M., Aiello M.O., Fujita M., Hinde K., Milligan L., Quinn E.A. (2013). Field and laboratory methods in human milk research. Am. J. Hum. Biol..

[139] Koc H., Mar M.H., Ranasinghe A., Swenberg J.A., Zeisel S.H. (2002). Quantitation of choline and its metabolites in tissues and foods by liquid chromatography/electrospray ionization-isotope dilution mass spectrometry. Anal. Chem..

[140] Phillips M.M. (2012). Analytical approaches to determination of total choline in foods and dietary supplements. Anal. Bioanal. Chem..

[141] Hampel D., Allen L.H. (2016). Analyzing B-vitamins in human milk: Methodological approaches. Crit. Rev. Food Sci. Nutr..

[142] World Health Organization and United Nations International Children’s Emergency (2008). Acceptable Medical Reasons for Use of Breastmilk Substitutes.

[143] Koletzko B., Baker S., Cleghorn G., Neto U.F., Gopalan S., Hernell O., Hock Q.S., Jirapinyo P., Lonnerdal B., Pencharz P. (2005). Global standard for the composition of infant formula: Recommendations of an ESPGHAN coordinated international expert group. J. Pediatr. Gastroenterol. Nutr..

[144] Codex Alimentarius Commission (2007). CODEX STAN 72-1981 for Infant Formula and Formulas for Special Medical Purposes Intended for Infants.

[145] Fu S., Tao B., Lai S., Zhang J., Ren Y.P. (2012). Determination of total choline by liquid chromatography-electrospray ionization-tandem mass spectrometry in infant formulas. AOAC Int..

[146] Pardini R.S., Sapien R.E. (2003). Trimethylaminuria (fish odor syndrome) related to the choline concentration of infant formula. Pediatr. Emerg. Care.

[147] Jing W., Thompson J.J., Jacobs W.A., Salvati L.M. (2015). Determination of free and total carnitine and choline in infant formulas and adult nutritional products by UPLC/MS/MS: Single-laboratory validation, first action 2014.04. AOAC Int..

[148] United Stated Department of Agriculture (2004). Database for the Choline Content of Common Foods, Release 1.

[149] Chern M.K., Gage D.A., Pietruszko R. (2000). Betaine aldehyde, betaine, and choline levels in rat livers during ethanol metabolism. Biochem. Pharmacol..

[150] Zeisel S.H., Mar M.H., Howe J.C., Holden J.M. (2003). Concentrations of choline-containing compounds and betaine in common foods. J. Nutr..

[151] Dilger R.N., Garrow T.A., Baker D.H. (2007). Betaine can partially spare choline in chicks but only when added to diets containing a minimal level of choline. J. Nutr..

[152] Lewis E.D., Kosik S.J., Zhao Y.Y., Jacobs R.L., Curtis J.M., Field C.J. (2014). Total choline and choline-containing moieties of commercially available pulses. Plant Foods Hum. Nutr..

[153] Lewis E.D., Zhao Y.Y., Richard C., Bruce H.L., Jacobs R.L., Field C.J., Curtis J.M. (2015). Measurement of the abundance of choline and the distribution of choline-containing moieties in meat. Int. J. Food Sci. Nutr..

[154] Hofvander Y., Hagman U., Hillervik C., Sjolin S. (1982). The amount of milk consumed by 1–3 months old breast- or bottle-fed infants. Acta Paediatr. Scand..

[155] Butte N.F., Garza C., Smith E.O., Nichols B.L. (1984). Human milk intake and growth in exclusively breast-fed infants. J. Pediatr..

[156] Widdowson E.M., McCance R.A. (1963). The effect of finite periods of undernutrition at different ages on the composition and subsequent development of the rat. Proc. R. Soc. Lond. B Biol. Sci..

[157] Welsch F. (1976). Studies on accumulation and metabolic fate of (*N*-Me^3^H)choline in human term placenta fragments. Biochem. Pharmacol..

[158] Pomfret E.A., daCosta K.A., Schurman L.L., Zeisel S.H. (1989). Measurement of choline and choline metabolite concentrations using high-pressure liquid chromatography and gas chromatography-mass spectrometry. Anal. Biochem..

[159] Pauwels S., Dopere I., Huybrechts I., Godderis L., Koppen G., Vansant G. (2015). Reproducibility and validity of an FFQ to assess usual intake of methyl-group donors. Public Health Nutr..

[160] Vennemann F.B., Ioannidou S., Valsta L.M., Dumas C., Ocke M.C., Mensink G.B., Lindtner O., Virtanen S.M., Tlustos C., D’Addezio L. (2015). Dietary intake and food sources of choline in European populations. Br. J. Nutr..

[161] Fischer L.M., daCosta K.A., Kwock L., Stewart P.W., Lu T.S., Stabler S.P., Allen R.H., Zeisel S.H. (2007). Sex and menopausal status influence human dietary requirements for the nutrient choline. Am. J. Clin. Nutr..

[162] Boyd W.D., Graham-White J., Blackwood G., Glen I., McQueen J. (1977). Clinical effects of choline in Alzheimer senile dementia. Lancet.

[163] Fischer L.M., da Costa K.A., Kwock L., Galanko J., Zeisel S.H. (2010). Dietary choline requirements of women: Effects of estrogen and genetic variation. Am. J. Clin. Nutr..

[164] Da Costa K.A., Kozyreva O.G., Song J., Galanko J.A., Fischer L.M., Zeisel S.H. (2006). Common genetic polymorphisms affect the human requirement for the nutrient choline. FASEB J..

[165] Veenema K., Solis C., Li R., Wang W., Maletz C.V., Abratte C.M., Caudill M.A. (2008). Adequate intake levels of choline are sufficient for preventing elevations in serum markers of liver dysfunction in Mexican American men but are not optimal for minimizing plasma total homocysteine increases after a methionine load. Am. J. Clin. Nutr..

[166] Kroke A., Klipstein-Grobusch K., Voss S., Moseneder J., Thielecke F., Noack R., Boeing H. (1999). Validation of a self-administered food-frequency questionnaire administered in the European prospective investigation into cancer and nutrition (EPIC) study: Comparison of energy, protein, and macronutrient intakes estimated with the doubly labeled water, urinary nitrogen, and repeated 24-h dietary recall methods. Am. J. Clin. Nutr..

[167] Yonemori K.M., Lim U., Koga K.R., Wilkens L.R., Au D., Boushey C.J., Le Marchand L., Kolonel L.N., Murphy S.P. (2013). Dietary choline and betaine intakes vary in an adult multiethnic population. J. Nutr..

[168] Thompson F.E., Byers T. (1994). Dietary assessment resource manual. J. Nutr..

[169] Cade J., Thompson R., Burley V., Warm D. (2002). Development, validation and utilisation of food-frequency questionnaires—A review. Public Health Nutr..

[170] Bingham S.A., Gill C., Welch A., Day K., Cassidy A., Khaw K.T., Sneyd M.J., Key T.J., Roe L., Day N.E. (1994). Comparison of dietary assessment methods in nutritional epidemiology: Weighed records v. 24 h recalls, food-frequency questionnaires and estimated-diet records. Br. J. Nutr..

[171] Block G. (1982). A review of validations of dietary assessment methods. Am. J. Epidemiol..

[172] Thompson R.L., Margetts B.M. (1993). Comparison of a food frequency questionnaire with a 10-day weighed record in cigarette smokers. Int. J. Epidemiol..

[173] Schatzkin A., Kipnis V., Carroll R.J., Midthune D., Subar A.F., Bingham S., Schoeller D.A., Troiano R.P., Freedman L.S. (2003). A comparison of a food frequency questionnaire with a 24-h recall for use in an epidemiological cohort study: Results from the biomarker-based observing protein and energy nutrition (OPEN) study. Int. J. Epidemiol..

[174] Basiotis P.P., Welsh S.O., Cronin F.J., Kelsay J.L., Mertz W. (1987). Number of days of food intake records required to estimate individual and group nutrient intakes with defined confidence. J. Nutr..

[175] Ma Y., Olendzki B.C., Pagoto S.L., Hurley T.G., Magner R.P., Ockene I.S., Schneider K.L., Merriam P.A., Hebert J.R. (2009). Number of 24-h diet recalls needed to estimate energy intake. Ann. Epidemiol..

[176] Lavery A.M., Brender J.D., Zhao H., Sweeney A., Felkner M., Suarez L., Canfield M.A. (2014). Dietary intake of choline and neural tube defects in Mexican Americans. Birth Defects Res. A Clin. Mol. Teratol..

[177] Du Y.F., Luo W.P., Lin F.Y., Lian Z.Q., Mo X.F., Yan B., Xu M., Huang W.Q., Huang J., Zhang C.X. (2016). Dietary choline and betaine intake, choline-metabolising genetic polymorphisms and breast cancer risk: A case-control study in china. Br. J. Nutr..

[178] Bidulescu A., Chambless L.E., Siega-Riz A.M., Zeisel S.H., Heiss G. (2007). Usual choline and betaine dietary intake and incident coronary heart disease: The atherosclerosis risk in communities (ARIC) study. BMC Cardiovasc. Disord..

[179] Cohen J. (1968). Weighted kappa: Nominal scale agreement with provision for scaled disagreement or partial credit. Psychol. Bull..

[180] Beaton G.H., Milner J., Corey P., McGuire V., Cousins M., Stewart E., de Ramos M., Hewitt D., Grambsch P.V., Kassim N. (1979). Sources of variance in 24-h dietary recall data: Implications for nutrition study design and interpretation. Am. J. Clin. Nutr..

[181] Willett W.C., Sampson L., Stampfer M.J., Rosner B., Bain C., Witschi J., Hennekens C.H., Speizer F.E. (1985). Reproducibility and validity of a semiquantitative food frequency questionnaire. Am. J. Epidemiol..

[182] Bland J.M., Altman D.G. (1986). Statistical methods for assessing agreement between two methods of clinical measurement. Lancet.

[183] Bland J.M., Altman D.G. (1999). Measuring agreement in method comparison studies. Stat. Methods Med. Res..

[184] Willett W. (2013). Nutritional Epidemiology.

[185] Cho E., Zeisel S.H., Jacques P., Selhub J., Dougherty L., Colditz G.A., Willett W.C. (2006). Dietary choline and betaine assessed by food-frequency questionnaire in relation to plasma total homocysteine concentration in the Framingham offspring study. Am. J. Clin. Nutr..

[186] Brunst K.J., Kannan S., Ni Y.M., Gennings C., Ganguri H.B., Wright R.J. (2016). Validation of a food frequency questionnaire for estimating micronutrient intakes in an urban US sample of multi-ethnic pregnant women. Matern Child Health J..

[187] Coathup V., Wheeler S., Smith L. (2016). A method comparison of a food frequency questionnaire to measure folate, choline, betaine, vitamin c and carotenoids with 24-h dietary recalls in women of reproductive age. Eur. J. Clin. Nutr..

[188] Fayet F., Flood V., Petocz P., Samman S. (2011). Relative and biomarker-based validity of a food frequency questionnaire that measures the intakes of vitamin B(12), folate, iron, and zinc in young women. Nutr. Res..

[189] Jackson K.A., Byrne N.M., Magarey A.M., Hills A.P. (2008). Minimizing random error in dietary intakes assessed by 24-h recall, in overweight and obese adults. Eur. J. Clin. Nutr..

[190] Willett W.C., Howe G.R., Kushi L.H. (1997). Adjustment for total energy intake in epidemiologic studies. Am. J. Clin. Nutr..

[191] Fischer L.M., Scearce J.A., Mar M.H., Patel J.R., Blanchard R.T., Macintosh B.A., Busby M.G., Zeisel S.H. (2005). Ad libitum choline intake in healthy individuals meets or exceeds the proposed adequate intake level. J. Nutr..

[192] Chester D.N., Goldman J.D., Ahuja J.K., Moshfegh A. Dietary Intakes of Choline: What We Eat in America, NHANES 2007–2008, 2011. www.ars.usda.gov/Services/docs.html?docid=19476.

[193] Wallace T.C., Fulgoni V.L. (2016). Assessment of total choline intakes in the United States. J. Am. Coll. Nutr..

[194] Wallace T.C., Fulgoni V.L. (2017). Usual choline intakes are associated with egg and protein food consumption in the United States. Nutrients.

[195] Gao X., Wang Y., Randell E., Pedram P., Yi Y., Gulliver W., Sun G. (2016). Higher dietary choline and betaine intakes are associated with better body composition in the adult population of Newfoundland, Canada. PLoS ONE.

[196] Lopez-Carrillo L., Gamboa-Loira B., Becerra W., Hernandez-Alcaraz C., Hernandez-Ramirez R.U., Gandolfi A.J., Franco-Marina F., Cebrian M.E. (2016). Dietary micronutrient intake and its relationship with arsenic metabolism in Mexican women. Environ. Res..

[197] Cheng C.P., Chen C.H., Kuo C.S., Kuo H.T., Huang K.T., Shen Y.L., Chang C.H., Huang R.F.S. (2017). Dietary choline and folate relationships with serum hepatic inflammatory injury markers in Taiwanese adults. Asia Pac. J. Clin. Nutr..

[198] Institute of Medicine (2000). Dietary Reference Intakes: Applications in Dietary Assessment.

[199] Nagata C., Wada K., Tamura T., Konishi K., Kawachi T., Tsuji M., Nakamura K. (2015). Choline and betaine intakes are not associated with cardiovascular disease mortality risk in Japanese men and women. J. Nutr..

[200] Mygind V.L., Evans S.E., Peddie M.C., Miller J.C., Houghton L.A. (2013). Estimation of usual intake and food sources of choline and betaine in New Zealand reproductive age women. Asia Pac. J. Clin. Nutr..

[201] Chiuve S.E., Giovannucci E.L., Hankinson S.E., Zeisel S.H., Dougherty L.W., Willett W.C., Rimm E.B. (2007). The association between betaine and choline intakes and the plasma concentrations of homocysteine in women. Am. J. Clin. Nutr..

[202] Cho E., Willett W.C., Colditz G.A., Fuchs C.S., Wu K., Chan A.T., Zeisel S.H., Giovannucci E.L. (2007). Dietary choline and betaine and the risk of distal colorectal adenoma in women. J. Natl. Cancer Inst..

[203] Xu X., Gammon M.D., Zeisel S.H., Bradshaw P.T., Wetmur J.G., Teitelbaum S.L., Neugut A.I., Santella R.M., Chen J. (2009). High intakes of choline and betaine reduce breast cancer mortality in a population-based study. FASEB J..

[204] Lee J.E., Giovannucci E., Fuchs C.S., Willett W.C., Zeisel S.H., Cho E. (2010). Choline and betaine intake and the risk of colorectal cancer in men. Cancer Epidemiol. Biomarkers Prev..

[205] Richman E.L., Kenfield S.A., Stampfer M.J., Giovannucci E.L., Zeisel S.H., Willett W.C., Chan J.M. (2012). Choline intake and risk of lethal prostate cancer: Incidence and survival. Am. J. Clin. Nutr..

[206] Lewis E.D., Subhan F.B., Bell R.C., McCargar L.J., Curtis J.M., Jacobs R.L., Field C.J., APrON Team (2014). Estimation of choline intake from 24 h dietary intake recalls and contribution of egg and milk consumption to intake among pregnant and lactating women in Alberta. Br. J. Nutr..

[207] Brunst K.J., Wright R.O., DiGioia K., Enlow M.B., Fernandez H., Wright R.J., Kannan S. (2014). Racial/ethnic and sociodemographic factors associated with micronutrient intakes and inadequacies among pregnant women in an urban US population. Public Health Nutr..

[208] Masih S.P., Plumptre L., Ly A., Berger H., Lausman A.Y., Croxford R., Kim Y.I., O’Connor D.L. (2015). Pregnant Canadian women achieve recommended intakes of one-carbon nutrients through prenatal supplementation but the supplement composition, including choline, requires reconsideration. J. Nutr..

[209] Malinowska A.M., Szwengiel A., Chmurzynska A. (2017). Dietary, anthropometric, and biochemical factors influencing plasma choline, carnitine, trimethylamine, and trimethylamine-*N*-oxide concentrations. Int. J. Food Sci. Nutr..

[210] Pauwels S., Ghosh M., Duca R.C., Bekaert B., Freson K., Huybrechts I., Langie S.A.S., Koppen G., Devlieger R., Godderis L. (2017). Maternal intake of methyl-group donors affects DNA methylation of metabolic genes in infants. Clin. Epigenetics.

[211] Jiang X., Bar H.Y., Yan J., Jones S., Brannon P.M., West A.A., Perry C.A., Ganti A., Pressman E., Devapatla S. (2013). A higher maternal choline intake among third-trimester pregnant women lowers placental and circulating concentrations of the antiangiogenic factor fms-like tyrosine kinase-1 (sflt1). FASEB J..

[212] American Medical Association (2017). Busines of the American Medical Association House of Delegates Annual Meeting. https://www.Ama-assn.Org/aboutus/business-ama-house-delegates-2017-annualmeeting#annotated%20reference%20committee%20reports.

[213] Wu B.T. (2014). Choline Nutrition, Choline Status, and Developmental Outcome in Early Childhood. Master’s Thesis.

[214] Reinhard P.C., Lotrean L.M. (2017). Choline intake and its food sources in the diet of Romanian kindergarten children. Nutrients.

[215] Al-Daghri N.M., Al-Othman A., Alkharfy K.M., Alokail M.S., Khan N., Alfawaz H.A., Aiswaidan I.A., Chrousos G.P. (2012). Assessment of selected nutrient intake and adipocytokine profile among Saudi children and adults. Endocr. J..

[216] Goon S., Dey S.R. (2014). A 24-h dietary recall for assessing the intake pattern of choline among Bangladeshi pregnant women at their third trimester of pregnancy. Cent. Asian J. Glob. Health.

[217] Gossell-Williams M., Fletcher H., McFarlane-Anderson N., Jacob A., Patel J., Zeisel S. (2005). Dietary intake of choline and plasma choline concentrations in pregnant women in Jamaica. West Indian Med. J..

